# A treatment for banknotes against viruses, bacteria and fungi

**DOI:** 10.1186/1753-6561-5-S6-O37

**Published:** 2011-06-29

**Authors:** FN Renaud, H Rosset, N Vast

**Affiliations:** 1 NOSOCO.TECH, UMR-CNRS-5510 MATEIS, Université Lyon1, Lyon, France; 2 Research & Development, Arjowiggins Security, Apprieu, France; 3 Marketing, Arjowiggins Security, Paris, France

## Introduction / objectives

Banknote paper was treated in order to prevent growth of micro organisms and consequently limit risks of contamination during handling.

## Methods

The Biogard√^®^ paper substrate was treated by impregnation with patented harmless active compounds. Antibacterial activity against *Eschericha coli* and *Staphylococcus aureus* was determined using ISO 20743 (transfer method) standard, antifungal activity using AATCC 30 (part III) against *Aspergillus niger* and antiviral activity using EN 1447666+A1 standard against H1H1 influenza A virus. Biocompatibility was assessed according to ISO 10993 standard with a skin irritation study in the rabbit and a sensitization study in the guinea pig.

## Results

Antibacterial activity: no CFU was observed for both species after 24 hours of incubation. In these conditions the bacteria could not grow on the paper and were even killed. Antifungal activity: no growth on the surface paper was observed after 7 days of incubation. *Aspergillus niger* development was totally inhibited.

Antiviral activity: the reduction rate of viral titre was greater than 4 (4.15) showing that the paper had a strong virucidal activity against H1N1 influenza A virus after a contact time of 1 hour.

## Conclusion

Banknotes are one of the most frequently-handled documents in the world. Most bacteria and fungi can survive a very long time on this kind of support. Even some virus can survive in specific environmental conditions. Moreover, it has been shown that banknotes can contribute to the transmission of pathogenic germs. The antimicrobial properties of Biogard√^®^ banknote treatment could be an innovative approach in infection control and contribute to preventing cross contaminations.

## Disclosure of interest

None declared.

